# Inositol Derivatives and Phenolic Compounds from the Roots of *Taraxacum coreanum*

**DOI:** 10.3390/molecules22081349

**Published:** 2017-08-14

**Authors:** Eun Jin Mo, Jong Hoon Ahn, Yang Hee Jo, Seon Beom Kim, Bang Yeon Hwang, Mi Kyeong Lee

**Affiliations:** College of Pharmacy, Chungbuk National University, Cheongju, Chungbuk 28160, Korea; mej2403@nate.com (E.J.M.); zzonggoo07@naver.com (J.H.A.); qow0125@naver.com (Y.H.J.); suntiger85@hanmail.net (S.B.K.); byhwang@chungbuk.ac.kr (B.Y.H.)

**Keywords:** *Taraxacum coreanum*, inositol, phenolic, antioxidant

## Abstract

In this study, the characterization of chemical constituents and biological activity of the roots of *Taraxacum coreanum* (Asteraceae) was attempted. Phytochemical investigation of the roots of *T. coreanum* led to the isolation of two new inositol derivatives, taraxinositols A (**1**) and B (**2**), and a new phenolic compound, taraxinol (**16**), together with twenty known compounds including four inositol derivatives, *neo*-inositol-1,4-bis (4-hydroxybenzeneacetate) (**3**), *chiro*-inositol-1,5-bis(4- hydroxybenzeneacetate) (**4**), *chiro*-inositol-2,3-bis (4-hydroxybenzeneacetate) (**5**) and *chiro*-inositol- 1,2,3-tris (4-hydroxybenzeneacetate) (**6**), nine phenolic compounds: *p*-hydroxybenzaldehyde (**7**), vanillin (**8**), syringaldehyde (**9**), vanillic acid (**10**), 4-methoxyphenylacetic acid (**11**), 4-hydroxy- phenylacetic acid methyl ester (**12**), optivanin (**13**), isoferulic acid (**14**) and dihydroconiferyl alcohol (**15**), four coumarins: nodakenetin (**17**), decursinol (**18**), prangol (**19**) and isobyakangelicin (**20**), and three lignans: syringaresinol-4′-*O*-β-d-glucoside (**21**), syringaresinol (**22**), and pinoresinol (**23**). The structures of isolated compounds were determined on the basis of spectroscopic analysis. Among the isolated compounds, vanillic acid, isoferulic acid and syringaresinol showed radical scavenging activity with IC_50_ values ranging from 30.4 to 75.2 μM.

## 1. Introduction

Plants of the genus *Taraxacum* are perennial herbs of the Asteraceae family. They are commonly called as dandelions and are widespread throughout the temperate climate regions. They are regarded as non-toxic edible herbs, and thus consumed as foods and food products. Recently they are also being for nutrition and medicinal purposes due to their diverse health-promoting effects [1]. Traditionally, they have been used to relieve inflammation and rheumatism [2]. Investigation of these species also revealed other potential beneficial effects such as antioxidant, neuroprotective, and hepatoprotective activities [3,4,5,6]. Phytochemical studies have reported the presence of diverse constituents from leaves, roots and flowers of *Taraxacum* species. Sesquiterpene lactones together with phenylpropanoids and terpenoids are known to be responsible for the diverse biological activities of dandelion [7,8,9,10,11]. 

Oxidative stress is well known for its harmful effects on health. It contributes to diverse diseases such as cancer, inflammation, neurodegenerative diseases and diabetes, as well as fatigue and senescence [12,13,14]. Although our body has defense systems against oxidative stress, overproduction of reactive oxygen species causes detrimental effects. Therefore, many investigators have been focused on the development of potent antioxidant materials. Plants are suggested as good candidates due to the diversity of compounds they contain [15,16,17]. 

In the present study, EtOAc and CH_2_Cl_2_ fractions of *T. coreanum* showed antioxidant activity in a DPPH radical scavenging assay. In a continuation of our research on bioactive natural products, extensive chromatographic separation was conducted for the isolation of constituents from *T. coreanum* roots. As a result, two new inositol derivatives, taraxinositols A (**1**) and B (**2**), and a new phenolic compound, taraxinol (**16**), were isolated from the roots of *T. coreanum,* together with twenty known compounds, including four inositol derivatives **3**–**6**, nine phenolic compounds **7**–**15**, four coumarins **17**–**20** and three lignans **21**–**23**. The antioxidant activity of the isolated compounds was also investigated.

## 2. Results

### 2.1. Structure Elucidation of the New Compounds

#### 2.1.1. Taraxinositol A (**1**)

**Figure 1:**
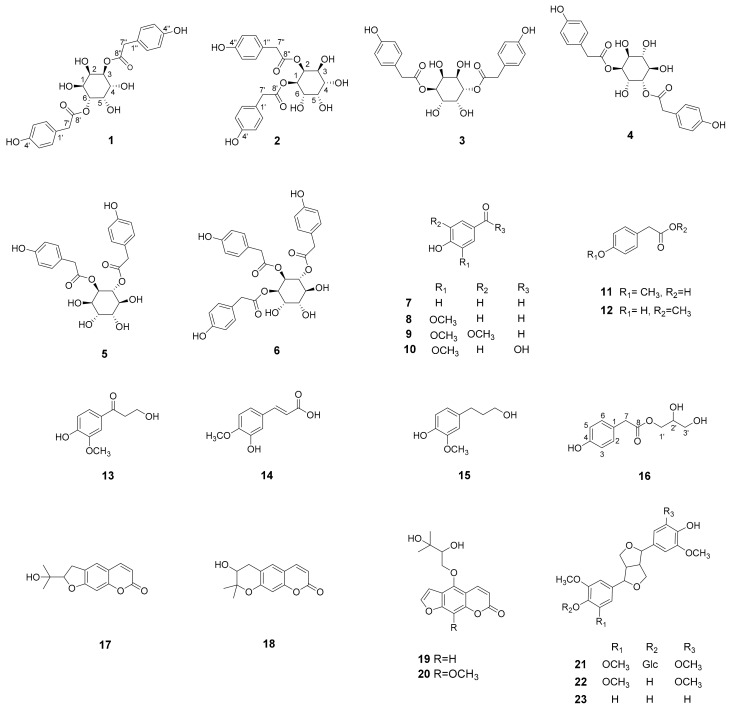
Chemical structures of compounds **1**–**23** from *T. coreanum*.

Compound **1** was obtained as a brown syrup and its molecular formula C_22_H_24_O_10_ was determined by an HRESIMS ion at *m*/*z* 471.1261 ([M + Na]^+^, calcd. 471.1267). Its IR spectrum showed absorption bands at 1685 and 3321 cm^−1^, indicating the presence of carbonyl and hydroxyl groups, respectively. Considering the HRESIMS molecular formula, the ^13^C-NMR spectrum only showed 11 carbon resonances, which suggested **1** as a symmetric structure. The presence of *p*-hydroxy-phenylacetic acid was deduced from the signals for 1,4-disubstituted aromatic rings at [δ_H_ 6.73 (4H, d, *J* = 8.5 Hz, H-3′, 5′, 3″, 5″), 7.03 (4H, d, *J* = 8.5 Hz, H-2′, 6′, 2″, 6″); δ_C_ 124.9 (C-1′, 1″), 130.1 (C-2′, 6′, 2″, 6″), 114.8 (C-3′, 5′, 3″, 5″), 156.1 (C-4′, 4″)], methylene signals at [δ_H_ 3.29 (2H, d, *J* = 15.5 Hz, H-7′a, 7″a), 3.41 (2H, d, *J* = 15.5 Hz, H-7′b, 7″b); δ_C_ 39.6 (C-7′, 7″)] and carbonyl signal at [δ_C_ 172.3 (C-8′, 8″)] in the ^1^H- and ^13^C-NMR spectrum, which was confirmed by HMBC correlations between H-7′/7″ and C-2′/2″, 8′/8″ and H-3′/3″ and C-4′/4″. Additionally, six hydroxymethine signals were observed at [δ_H_ 3.93 (2H, dt, *J* = 7.0. 2.5 Hz, H-1, 4), 3.95 (2H, d, *J* = 2.0 Hz, H-2, 5), 5.22 (2H, dd, *J* = 7.0. 2.5 Hz, H-3, 6); δ_C_ 69.1 (C-1, 4), 72.1 (C-2, 5), 73.3 (C-3, 6)], which suggested **1** was an inositol derivative [7]. The relatively large coupling constant of 7.0 Hz (*J*_1,6_, *J*_3,4_) suggested two pairs of *trans*-axial protons (H-1, 6 and H-3,4). On the other hand, the vicinal axial-equatorial protons (H-1,2, H-2,3, H-4,5 and H-5,6) showed small coupling constants of less than 3 Hz. Based on the multiplicities and coupling constants, the stereochemistry of **1** has been deduced as a neo-inositol [3]. Linkages of the *p*-hydroxyphenylacetic acid moieties to the inositol unit were determined from HMBC correlations between H-3/6 and C-8′/8″. In addition, chemical shift of H-3/6 (δ_H_ 5.22) was downfield shifted compared to H-1/4 and H-2/5, which is the characteristic of ester bond of alcohol moiety. Taken together, the structure of **1** was determined to be as shown in Figure 1 and the new compound was named taraxinositol A.

#### 2.1.2. Taraxinositol B (**2**)

Compound **2** was purified as a brown syrup with a molecular formula of C_22_H_24_O_10_ from the HRESIMS ion at *m*/*z* 471.1261 ([M + Na]^+^, calcd. 471.1267) and ^13^C-NMR data. The ^1^H- and ^13^C-NMR patterns were quite similar to those of **1**, but the ^13^C-NMR spectrum now showed 22 carbon resonances which suggested **2** as an asymmetric inositol derivative. The presence of the inositol moiety was shown by peaks at [δ_H_ 5.09 (1H, dt, *J* = 7.0, 3.5 Hz, H-1), 5.29 (1H, t, *J* = 3.5 Hz, H-2), 3.55 (1H, dd, *J* = 9.5, 3.0 Hz, H-3), 3.66 (1H, dd, *J* = 9.5, 2.5 Hz, H-4), 3.84 (1H, t, *J* = 3.5 Hz, H-5), 3.66 (1H, dd, *J* = 7.0, 2.5 Hz, H-6); δ_C_ 72.2 (C-1), 70.9 (C-2), 71.0 (C-3), 71.1 (C-4), 69.5 (C-5), 72.9 (C-6)]. The stereochemistry of the inositol moiety has been also determined based on the multiplicities and coupling constants. Two pairs of *trans*-axial protons (H-1,6 and H-3,4) with large coupling constants of >7.0 Hz (*J*_1,6_, *J*_3,4_) and additional vicinal axial-equatorial protons (H-1,2, H-2,3, H-4,5 and H-5,6) with relatively small coupling constants of less than 3 Hz suggested **2** is also a neo-inositol [3]. Besides the abovementioned inositol moiety signals, the two *p*-hydroxyphenylacetic acids were deduced from the signals of 1,4-disubstituted aromatic rings at [δ_H_ 7.02 (2H, d, *J* = 8.5 Hz, H-2′, 6′), 6.72 (2H, d, *J* = 8.5 Hz, H-3′, 5′), 7.12 (2H, d, *J* = 8.5 Hz, H-2″, 6″), 6.77 (2H, d, *J* = 8.5 Hz, H-3″, 5″); δ_C_ 124.7 (C-1′, 1″), 130.1 (C-2′, 6′, 2″, 6″), 115.0 (C-3′, 5′, 3″, 5″), 156.0 (C-4′), 156.3 (C-4″)], methylene signals at [δ_H_ 3.55 (1H, s, H-7′a), 3.41 (1H, s, H-7′b), 3.50 (2H, d, *J* = 2.5 Hz, H-7″b); δ_C_ 39.4 (C-7′), 39.9 (C-7″)] and carbonyl signal at [δ_C_ 172.1 (C-8′), 171.1 (C-8″)] in the ^1^H and ^13^C-NMR spectrum together with HMBC correlations. Linkages of the *p*-hydroxyphenylacetic acid moieties to C-1 and C-2 of the inositol unit were determined from HMBC correlations of H-1 to C-8′ and H-2 to C-8″, which was confirmed by the chemical shift of H-1 (δ_H_ 5.09) and H-2 (δ_H_ 5.29). Taken together, the structure of **2** was determined as shown in Figure 1 and the compound was named taraxinositol B.

#### 2.1.3. Taraxinol (**16**)

**Figure 2:**
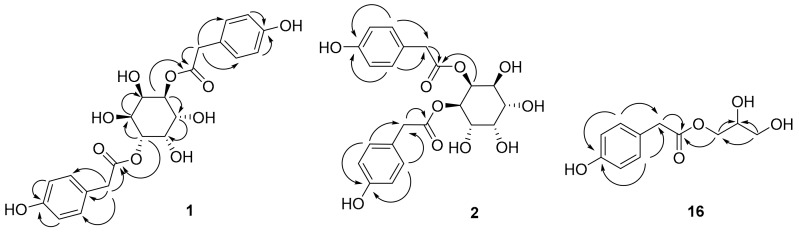
Key HMBC correlations of compounds **1**, **2** and **16**.

Compound **16** was purified as a colorless syrup and showed an HRESIMS ion at *m*/*z* 249.0733 ([M + Na]^+^, calcd. 249.0739) for C_11_H_14_O_5_Na. The ^1^H- and ^13^C-NMR spectra of **16** showed resonances for 1,4-disubstituted aromatic rings at [δ_H_ 7.12 (2H, d, *J* = 8.8 Hz, H-2, 6) and 6.74 (2H, d, *J* = 8.8 Hz, H-3,5); δ_C_ 124.9 (C-1), 130.0 (C-2, 6), 114.9 (C-3, 5), and 156.2 (C-4)], one methylene at [δ_H_ 3.58 (2H, s, H-7); δ_C_ 39.6 (C-7)] and a carbonyl signal at δ_C_ 172.6 (C-8), which suggested the presence of a *p*-hydroxyphenylacetic acid. Additionally two hydroxymethylenes at [δ_H_ 4.09 (1H, dd, *J* = 11.2, 6.0 Hz, H-1′a), 4.17 (1H, dd, *J* = 11.6, 4.4 Hz, H-1′b); δ_C_ 65.4 (C-1′)] and [δ_H_ 3.54 (2H, dd, *J* = 5.2, 2.4 Hz, H-3′); δ_C_ 62.6 (C-1′)], and one hydroxymethine at [δ_H_ 3.83 (1H, m, H-2′); δ_C_ 69.7 (C-2′)] were observed in the ^1^H- and ^13^C-NMR spectrum. The HMBC correlations of H-1′ to C-2′ and H-3′ to C-1′ and C-2′, suggested the presence of partial structure of -CH_2_-CH(OH)-CH_2_OH. Further additional HMBC correlation of H-1′ to C-8 confirmed the connection of -CH_2_-CH(OH)-CH_2_OH to the C-8 of *p*-hydroxyphenylacetic acid. Thus, the structure of compound **16** was elucidated as shown in Figure 2 and the compound was given the common name taraxinol.

### 2.2. Identification of Known Compounds

Twenty known compounds were identified as *neo*-inositol-1,4-bis (4-hydroxybenzeneacetate) (**3**) [7], *chiro*-inositol-1,5-bis (4-hydroxybenzeneacetate) (**4**) [8], *chiro*-inositol-2,3-bis (4-hydroxy-benzeneacetate) (**5**) [6], *chiro*-inositol-1,2,3-tris (4-hydroxybenzeneacetate) (**6**) [6], *p*-hydroxy-benzaldehyde (**7**) [17], vanillin (**8**) [18], syringaldehyde (**9**) [17], vanillic acid (**10**) [18], 4-methoxyphenylacetic acid (**11**) [19], 4-hydroxyphenylacetic acid methyl ester (**12**) [20], optivanin (**13**) [21], isoferulic acid (**14**) [22], dihydroconiferyl alcohol (**15**) [23], nodakenetin (**17**) [24], decursinol (**18**) [25], prangol (**19**) [26], isobyakangelicin (**20**) [27], syringaresinol-4′-*O*-β-d-glucoside (**21**) [28], syringaresinol (**22**) [29], pinoresinol (**23**) [30] by spectroscopic analysis and comparison with reported data.

### 2.3. Antioxidative Activity of Isolated Compounds

Oxidative stress is known as a major contributor to diverse diseases and age-related symptoms [11,12,13], therefore, development of natural products with high antioxidant potential has been conducted for reducing harmful oxidative stress and further health promoting effect [14,15,16]. 

In our present study, the EtOAc and CH_2_Cl_2_ fraction of *T. coreanum* roots showed antioxidant activity in DPPH radical scavenging assay (36.9% and 56.8% inhibition, respectively, at 100 μg/mL). Further assessment of antioxidant activity of isolated compounds from *T. coreanum* demonstrated antioxidant activity of compounds **10**, **14** and **21**–**23**, whereas other compounds showed little effects. Compounds **10**, **14** and **22** showed IC_50_ values of 30.3, 34.6 and 75.4 μM, respectively. 

*Taraxacum* species have been reported to have diverse biological activity. Consistent with our present study, the antioxidant activity of the extract has been reported [3,31]. Besides, anti-inflammatory, antimicrobial, hepatoprotective and neuroprotective activities also have been demonstrated [4,5,6,32]. As active constituents, sesquiterpenoids and phenolic compounds were suggested as active principles of *Taraxacum* species [33,34,35].

Our present study showed the presence of diverse phenolic constituents in the roots of dandelions. Interestingly, phenolic compounds were found as inositol esters in the roots of *Taraxacum* species [36,37,38]. Inositols are cyclohexane-based carbocyclic polyols with six hydroxyl groups and nine isomers of inositol including are distributed in nature. Anti-inflammatory and anti-diabetic activity of inositol derivatives were also reported [39,40]. In our present study, we report six characteristic inositol derivatives including two new ones. Our present study also showed that lignans and phenolic compounds were antioxidant principles of *T. coreanum.* Inositols isolated in our present study, however, exerted weak activity in DPPH radical scavenging activity. Taken together, new constituents were characterized from *T. coreanum* roots and further investigation is needed for evaluation of the biological activity of these constituents. 

## 3. Materials and Methods

### 3.1. General Information

NMR spectra were recorded on a DRX 400 or 500 MHz NMR spectrometer (Bruker, Karlsruhe, Germany). ESI-mass spectra were obtained on a VG Autospec Ultima mass spectrometer (Waters, Milford, MA, USA). Semipreparative HPLC was performed on a HPLC system equipped with Waters 600 Q-pumps, a 996 photodiode array detector, and Waters Empower software (Waters, Milford, MA, USA) using a Gemini-NX ODS-column (5 μm, 10 × 150 mm). Silica gel (70−230 mesh, Merck, Darmstadt, Germany) and Sephadex LH-20 (25−100 μm, Amersham Biosciences, Uppsala, Sweden) were used for open column chromatography. Thin-layer chromatography (TLC) was performed on precoated silica gel 60 F_254_ plates (0.25 mm, Merck). All other chemicals and reagents were analytical grade.

### 3.2. Isolation of Compounds

The roots of *T. coreanum* were obtained from the local herbal market in Chungbuk (Korea) in April 2015 and were identified by the herbarium staff of the College of Pharmacy at Chungbuk National University, where a voucher specimen was deposited (CBNU201504-TC). The dried roots of *T. coreanum* (5.0 kg) were extracted two times with 80% MeOH (64 L), which yielded after solvent removal the total extract (1.2 kg). The total extract was then suspended in H_2_O (2.4 L). Further successive partitioning with *n*-hexane, CH_2_Cl_2_, EtOAc and *n*-BuOH (2.0 L each) yielded the *n*-hexane (27.8 g), CH_2_Cl_2_ (8.5 g), EtOAc (8.8 g) and *n*-BuOH (54.7 g) soluble fractions, respectively.

The CH_2_Cl_2_ fraction (8.5 g) was subjected to silica gel column chromatography eluting with a mixture of CH_2_Cl_2_–MeOH to give 12 fractions (M1–M12). M5 was subjected to silica gel column chromatography with a mixture of *n*-hexane–EtOAc to give 14 fractions (M5A–M5N). Compounds **7** (5.1 mg) and **8** (2.4 mg) were obtained from M5E and compounds **9** (3.1 mg) and **15** (1.6 mg) from M5I by semipreparative HPLC eluting with CH_3_CN–H_2_O. M5K was subjected to column chromatography over Sephadex LH-20 eluting with CH_2_Cl_2_–MeOH (1:1) to give six fractions (M5K1–M5K6). Compounds **17** (2.4 mg), **18** (2.0 mg) and **23** (2.7 mg) were purified from M5K3 by semipreparative HPLC eluting with CH_3_CN–H_2_O. Compounds **19** (8.3 mg) and **20** (11.6 mg) were purified from M5L by semipreparative HPLC eluting with CH_3_CN–H_2_O. Compound **21** (2.1 mg) and **22** (1.5 mg) were purified from M7 and M5M, respectively, by Sephadex LH-20 column chromatography with MeOH, followed by semipreparative HPLC eluting with CH_3_CN–H_2_O.

The EtOAc fraction (8.8 g) was subjected to silica column chromatography with a mixture of *n*-hexane–EtOAc–MeOH as eluent to give 11 fractions (E1–E11). Semipreparative HPLC of E4 eluting with CH_3_CN–H_2_O yielded compounds **10** (8.0 mg) and **14** (9.0 mg). E9 was subjected to RP-silica column chromatography with the mixture of MeOH–H_2_O to give 8 fractions (E9A–E9H). Compound **16** (14.1 mg) was obtained from E9B by semipreparative HPLC eluting with CH_3_CN–H_2_O. E9D was further subjected to column chromatography over silica gel eluting with CH_2_Cl_2_-MeOH to afford four subfractions (E9D1-E9D4). Compound **12** (19.9 mg) was obtained from E9D1 by semipreparative HPLC eluting with CH_3_CN–H_2_O. Compounds **1** (1.4 mg) and **6** (1.8 mg) were purified from E9D3 by semipreparative HPLC eluting with CH_3_CN-MeOH-H_2_O. E9F was subjected to RP-silica column chromatography with MeOH–H_2_O to give 6 fractions (E9F1–E9F6). Compounds **3** (1.3 mg) and **4** (4.2 mg) were purified from E9F5 by semipreparative HPLC eluting with CH_3_CN–H_2_O. E9G was subjected to RP-silica column chromatography with MeOH-H_2_O to give 5 fractions (E9G1–E9G5). Compounds **2** (6.7 mg) and **5** (4.5 mg) were purified from E9G2 by semipreparative HPLC eluting with CH_3_CN–H_2_O. 

Taraxinositol A (**1**) brown syrup; ${\left[\alpha \right]}_{D}^{25}$ +9.3 (*c* 0.03, MeOH); IR_max_ 1729, 3321 cm^−1^; ^1^H-NMR (500 MHz, CD_3_OD) and ^13^C-NMR (125 MHz, CD_3_OD) see Table 1; ESI-MS (positive mode) *m*/*z*: 471 [M + Na]^+^, HRESIMS (positive mode) *m*/*z*: 471.1261 [M + Na]^+^ (Calcd. for C_22_H_24_NaO_5_, 471.1267).

Taraxinositol B (**2**) brown syrup; ${\left[\alpha \right]}_{D}^{25}$ −13.5 (*c* 0.03, MeOH); IR_max_ 1763, 3367 cm^−1^; ^1^H-NMR (500 MHz, CD_3_OD) and ^13^C-NMR (125 MHz, CD_3_OD) see Table 1; ESI-MS (positive mode) *m*/*z*: 471 [M + Na]^+^, HRESIMS (positive mode) *m*/*z*: 471.1261 [M + Na]^+^ (Calcd. for C_22_H_24_NaO_5_, 471.1267).

Taraxinol (**16**): brown syrup; ${\left[\alpha \right]}_{D}^{25}$ −28.0 (*c* 0.01, MeOH); IR_max_ 1716, 3285 cm^−1^; ^1^H-NMR (400 MHz, CD_3_OD) and ^13^C-NMR (100 MHz, CD_3_OD) see Table 2; ESI-MS (positive mode) *m*/*z*: 249 [M + Na]^+^, HREIMS (positive mode) *m*/*z*: 249.0733 [M + Na]^+^ (Calcd. for C_11_H_14_ NaO_5_, 249.0739).

### 3.3. Evaluation of Antioxidant Activity

The antioxidant activity was evaluated by measuring the free radical scavenging activity using a DPPH assay. Briefly, samples were mixed with freshly prepared DPPH solution. After shaking, the reaction mixtures were stand for 30 min at room temperature in dark places. The radical scavenging activity was determined by measuring the absorbance at 517 nm. The relative radical scavenging activity (%) was calculated as [1 − absorbance of solution with sample and DPPH/absorbance of solution with DPPH] × 100.

## Figures and Tables

**Table 1:** ^1^H- and ^13^C-NMR spectroscopic data for compounds **1** and **2**.

Carbon No.	1		Carbon No.	2	
	^1^H	^13^C		^1^H	^13^C
1, 4	3.93 (2H, dt, *J =* 7.0, 2.5 Hz)	69.1	1	5.09 (1H, dt, *J =* 6.5, 3.5 Hz)	72.2
2, 5	3.95 (2H, d, *J =* 2.0 Hz)	72.1	2	5.29 (1H, t, *J =* 3.5 Hz)	70.9
3, 6	5.22 (2H, dd, *J =* 7.0, 2.5 Hz)	73.3	3	3.55 (1H, dd, *J =* 9.5, 3.0 Hz)	71.0
			4	3.66 (1H, dd, *J =* 9.5, 2.5 Hz)	71.1
			5	3.84 (1H, t, *J =* 3.5 Hz)	69.5
			6	3.66 (1H, d, *J =* 6.5, 2.5 Hz)	72.9
1′, 1″	-	124.9	1′	-	124.7
2′, 6′, 2″, 6″	7.03 (4H, d, *J =* 8.5 Hz)	130.1	2′, 6′	7.02 (1H, d, *J =* 8.5 Hz)	130.1
3′, 5′, 3″, 5″	6.73 (4H, d, *J =* 8.5 Hz)	114.8	3′, 5′	6.72 (1H, d, *J =* 8.5 Hz)	115.0
4′, 4″	-	156.1	4′	-	156.0
7′, 7″	3.29 (2H, d, *J =* 15.5 Hz)3.41 (2H, d, *J =* 15.5 Hz)	39.6	7′	3.55 (1H, s) 3.41 (1H, s)	39.4
8′, 8″	-	172.3	8′	-	172.1
			1″	-	124.7
			2″, 6″	7.12 (1H, d, *J =* 8.5 Hz)	130.1
			3″, 5″	6.77 (1H, d, *J =* 8.5 Hz)	115.0
			4″	-	156.3
			7″	3.50 (2H, d, *J =* 2.5 Hz)	39.9
			8″	-	171.1

**Table 2:** ^1^H- and ^13^C-NMR spectroscopic data for compound **16**.

Carbon No.	16	
	^1^H	^13^C
1	-	124.9
2, 6	7.12 (2H, d, *J =* 8.8 Hz)	130.0
3, 5	6.74 (2H, d, *J =* 8.4 Hz)	114.9
4	-	156.2
7	3.58 (2H, s)	39.6
8	-	172.6
1′	4.09 (1H, dd, *J =* 11.2, 6.0 Hz) 4.17 (1H, dd, *J =* 11.6, 4.4 Hz)	65.4
2′	3.83 (1H, m)	69.7
3′	3.54 (2H, dd, *J =* 5.2, 2.4 Hz)	62.6

## Supplementary Materials

Supplementary materials are available online.
