# Supplementary material for: Inositol Derivatives and Phenolic Compounds from the Roots of Taraxacum coreanum
Source: Molecules. 2017 Aug 14;22(8):1349. doi: 10.3390/molecules22081349 (PMC6152297; doi:10.3390/molecules22081349)
Supplement: Supplementary file 1 [file molecules-22-01349-s001.pdf]

Supplementary material

Inositol Derivatives and Phenolic Compounds from the Roots of *Taraxacum coreanum*

Eun Jin Mo, Jong Hoon Ahn, Yang Hee Jo, Seon Beom Kim, Bang Yeon Hwang and Mi Kyeong Lee*

College of Pharmacy, Chungbuk National University, Cheongju, Chungbuk 28160, Korea; mej2403@nate.com (E.J.M.); zzonggoo07@naver.com (J.H.A.); qow0125@naver.com (Y.H.J.); suntiger85@hanmail.net (S.B.K.); byhwang@chungbuk.ac.kr (B.Y.H.)

* Correspondence: mklee@chungbuk.ac.kr; Tel.: +82-43-261-2818

Table S1. ^{1}H and ^{13}C NMR spectroscopic data for compound **3**

Carbon No	**3**	
	^{1}H	^{13}C
1, 4	5.08 (2H, dt, *J* = 7.5, 2.0 Hz)	74.2
2, 5	3.88 (2H, dd, *J* = 7.0, 3.0 Hz)	71.1
3, 6	4.03 (2H, d, *J* = 2.0 Hz)	69.9
1′, 1′′	-	125.7
2′, 6′, 2′′, 6′′	7.14 (4H, d, *J* = 8.5 Hz)	130.1
3′, 5′, 3′′, 5′′	6.74 (4H, d, *J* = 8.5 Hz)	114.8
4′, 4′′	-	156.1
7′, 7′′	3.65 (4H, s)	39.5
8′, 8′′	-	172.5

Table S2. ^{1}H spectroscopic data for compounds **4-6**

Carbon No	**4**	**5**	**6**
1	4.82 (1H, dd, *J* = 10.5, 3.5 Hz)	4.04 (1H, t, *J* = 3.5 Hz)	5.28 (1H, t, *J* = 3.5 Hz)
2	3.83 (1H, t, *J* = 9.5 Hz)	5.13 (1H, dd, *J* = 10.5, 3.0)	5.15 (1H, dd, *J* = 10.5, 3.0 Hz)
3	3.57 (1H, t, *J* = 9.5 Hz)	5.33 (1H, t, *J* = 9.5 Hz)	5.25 (1H, t, *J* = 10.0 Hz)
4	3.92 (1H, dd, *J* = 10.0, 3.5 Hz)	3.76 (1H, t, *J* = 9.5 Hz)	3.80 (1H, t, *J* = 10.0 Hz)
5	5.15 (1H, t, *J* = 4.0 Hz)	3.81 (1H, dd, *J* = 10.0, 3.0 Hz)	3.62 (1H, dd, *J* = 10.0, 3.0 Hz)
6	3.99 (1H, t, *J* = 4.0 Hz)	3.94 (1H, t, *J* = 3.5 Hz)	3.84 (1H, t, *J* = 3.5 Hz)
2′, 6′	7.12 (2H, d, *J* = 8.5 Hz)	7.04 (2H, d, *J* = 8.5 Hz)	6.85 (2H, d, *J* = 8.0 Hz)
2″ 6″	7.14 (2H, d, *J* = 8.5 Hz)	7.07 (2H, d, *J* = 8.5 Hz	7.10 (2H, d, *J* = 8.5 Hz),
2‴, 6‴	-		7.14 (2H, d, *J* = 8.5 Hz)
3′, 5′	6.75 (2H, d, *J* = 9.0 Hz)	6.73 (4H, d, *J* = 8.5 Hz)	6.69 (2H, d, *J* = 8.5 Hz)
3″, 5″	6.73 (2H, d, *J* = 8.5 Hz)		6.75 (2H, d, *J* = 8.0 Hz)
3‴, 5‴	-	-	6.77 (2H, d, *J* = 8.5 Hz)
7′	3.65 (2H, m)	3.37 (4H, s)	3.50 (1H, s), 3.48 (1H, s)
7″	3.61 (2H, m),		3.44 (2H, d, *J* = 2.5 Hz)
7‴	-	-	2.86-2.96 (2H, m)

Recorded at 500MHz in CD_3OD.

Table S3. ^{1}H spectroscopic data for compounds **7-12**

Carbon No	**7**[a]	**8**[b]	**9**[a]	**10**[a]	**11**[b]	**12**[b]
1	-	-	-	-	-	
2	7.79 (1H, d, *J* = 8.0 Hz)	7.44 (1H, m)	7.25 (1H, s)	7.43 (1H, m)	6.96 (1H, d, *J* = 8.5 Hz)	7.09 (1H, d, *J* = 8.5 Hz)
3	6.93 (1H, d, *J* = 8.4 Hz)	-	-	-	6.84 (1H, d, *J* = 8.5 Hz)	6.74 (1H, d, *J* = 8.5 Hz)
4	-	-	-	-	-	-
5	6.93 (1H, d, *J* = 8.4 Hz)	6.95 (1H, d, *J* = 8.5 Hz)	-	6.81 (1H, d, *J* = 8.8 Hz)	6.84 (1H, d, *J* = 8.5 Hz)	6.74 (1H, d, *J* = 8.5 Hz)
6	7.79 (1H, d, *J* = 8.0 Hz)	7.44 (1H, m)	7.25 (1H, s)	7.43 (1H, m)	6.96 (1H, d, *J* = 8.5 Hz)	7.09 (1H, d, *J* = 8.5 Hz)
7	9.78 (1H, s)	9.75 (1H, s)	9.76 (1H, s)	-	3.49 (2H, s)	3.54 (2H, s)
8					-	-
$-OCH_3$		3.94 (3H, s)	3.94 (6H, s)	3.84 (3H, s)	3.74 (3H, s)	3.68 (3H, s)

[a] Recorded at 400MHz in CD_3OD, [b] Recorded at 500MHz in CD_3OD.

Table S4. ^{1}H spectroscopic data for compounds **13-15**

Carbon No	**13**[a]	**14** [a]	**15** [b]
1	-	-	-
2	7.57 (1H, d, *J* = 2.0 Hz)	7.05 (1H, d, *J* = 2.0 Hz)	6.79 (1H, d, *J* = 2.0 Hz)
3	-	-	-
4	-	-	-
5	6.89 (1H, d, *J* = 8.4 Hz)	6.79 (1H, d, *J* = 8.0 Hz)	6.71 (1H, d, *J* = 8.0 Hz)
6	7.60 (1H, d, *J* = 8.4, 2.0 Hz)	6.96 (1H, dd, *J* = 8.0, 2.0 Hz)	6.64 (1H, dd, *J* = 8.0, 2.0 Hz)
7	-	7.56 (1H, d, *J* = 16.0 Hz)	2.61 (2H, t, *J* = 7.5 Hz)
8	3.18 (2H, t, *J* = 6.4 Hz)	6.28 (1H, d, *J* = 16.0 Hz)	1.82 (2H, m)
9	3.96 (2H, t, *J* = 6.4 Hz)	-	3.57 (2H, t, *J* = 6.5 Hz)
-OCH_3	3.93 (3H, s)	3.77 (3H, s)	3.80 (3H, s)

a Recorded at 400MHz in CD_3OD, **b** Recorded at 500MHz in CD_3OD.

Table S5. ^{1}H spectroscopic data for compounds **17** and **18**

Carbon No	**17**	**18**
3	6.21 (1H, d, *J* = 9.6 Hz)	6.22 (1H, d, *J* = 9.2 Hz)
4	7.87 (1H, d, *J* = 9.6 Hz)	7.85 (1H, d, *J* = 9.2 Hz)
5	7.42 (1H, s)	7.37 (1H, s)
8	6.74 (1H, s)	6.72 (1H, s)
2′	4.78 (1H, t, *J* = 8.4 Hz)	
3′	3.27 (2H, m)	3.83 (1H, q, *J* = 6.8 Hz)
4′		3.12 (1H, dd, *J* = 16.8, 5.2 Hz)
		2.81 (1H, dd, *J* = 16.8, 6.8 Hz)
5′	1.31 (3H, s)	1.38 (3H, s)
6′	1.25 (3H, s)	1.34 (3H, s)

Recorded at 400MHz in CD_3OD.

Table S6. ^1H spectroscopic data for compounds **19** and **20**

Carbon No	**19**	**20**
3	6.30 (1H, d, *J* = 10.0 Hz)	6.30 (1H, d, J = 10.0 Hz)
4	8.44 (1H, d, *J* = 9.6 Hz)	8.24 (1H, d, J = 10.0 Hz)
8	7.21 (1H, s)	
2′	7.80 (1H, d, *J* = 2.4 Hz)	7.84 (1H, d, J = 2.4 Hz)
3′	7.24 (1H, d, *J* = 2.4 Hz)	7.23 (1H, d, J = 2.4 Hz)
1″	4.81 (1H, dd, *J* = 10.0, 2.4 Hz)	4.58 (1H, dd, J = 10.4, 2.8 Hz)
	4.41 (1H, q, *J* = 9.6 Hz)	4.30 (1H, q, J = 10.4, 8.0 Hz)
2″	3.84 (1H, dd, *J* = 8.8, 2.8 Hz)	3.85 (1H, dd, J = 8.0, 2.8 Hz)
4″	1.31 (3H, s)	1.28 (3H, s)
5″	1.26 (3H, s)	1.24 (3H, s)
-OCH_3		4.22 (3H, s)

Recorded at 400MHz in CD_3OD.

Table S7. ^{1}H spectroscopic data for compounds **21 - 23**

Carbon No	**21**	Carbon No	**22**	**23**
2, 6	6.74 (2H, s)	2, 2′	6.68 (2H, s)	6.97 (2H, d, *J* = 1.6 Hz)
7	4.79 (1H, d, *J* = 4.0 Hz)	5, 5′	-	6.79 (2H, d, *J* = 8.0 Hz)
8	3.15 (1H, m)	6, 6′	6.68 (2H, s)	6.84 (2H, dd, *J* = 8.4, 1.6 Hz)
9	3.93 (2H, dd, *J* = 9.2, 3.2 Hz)	7, 7′	4.74 (2H, d, *J* = 4.4 Hz)	4.73 (2H, d, *J* = 4.4 Hz)
2′, 6′	6.68 (2H, s)	8, 8′	3.17 (1H, m)	3.17 (1H, m)
7′	4.74 (1H, d, *J* = 4.4 Hz)	9, 9′	3.90 (2H, m)	3.86 (2H, m), 4.25 (2H, m)
8′	3.15 (1H, m)		4.29 (2H, m)	
9′	4.30 (2H, m)	3,3′–OCH_3	3.87 (6H, s)	3.88 (6H, s)
1″	4.80 (1H, s)	5,5′–OCH_3	3.87 (6H, s)	-
2″	3.50 (1H, m)			
3″	3.42 (1H, d, *J* = 2.8 Hz)			
4″	3.44 (1H, d, *J* = 2.8 Hz)			
5″	3.22 (1H, m)			
6″	3.80 (1H, dd, *J* = 12.0, 2.4 Hz)<br>3.68 (1H, dd, *J* = 12.0, 5.2 Hz)			
3,5–OCH_3	3.88 (6H, s)			
3′,5′–OCH_3	3.86 (6H, s)			

Recorded at 400MHz in CD_3OD.

Table S8. Antioxidant activity of compounds **1-23**.

Compounds	IC_{50} (μM)	Compounds	IC_{50} (μM)
**1**	>100	**13**	>100
**2**	>100	**14**	34.6
**3**	>100	**15**	>100
**4**	>100	**16**	>100
**5**	>100	**17**	>100
**6**	>100	**18**	>100
**7**	>100	**19**	>100
**8**	>100	**20**	>100
**9**	>100	**21**	81.6
**10**	30.4	**22**	75.4
**11**	>100	**23**	89.2
**12**	>100	quercetin [a]	19.8

[a] Quercetin was used as the positive control.
